# Dataset on stand structure in hemiboreal forests across management histories and stand ages

**DOI:** 10.1016/j.dib.2026.112688

**Published:** 2026-03-18

**Authors:** Raul Rosenvald, Asko Lõhmus, Raido Kont, Ann Kraut, Piret Lõhmus, Liina Remm, Triin Tekko, Maarja Vaikre, Kadri Runnel

**Affiliations:** Institute of Ecology and Earth Sciences, University of Tartu, J. Liivi 2, 50409 Tartu, Estonia

**Keywords:** Boreo-nemoral, Clear-cut, Dead wood volume, Old-growth forest, Thinning, Timber stock, Tree species diversity

## Abstract

**Data description:**

This dataset compiles detailed representative forest stand structure data from a European hemiboreal forest region; it spans multiple forest types, successional stages, and forest management contexts. It integrates measurements of live trees and dead wood, including fallen trunks, snags (standing dead trees), stumps and fine woody debris (down to 3 mm diameter), together with tree species identity, dimensional attributes, and decay stages. A subset of stands has been surveyed repeatedly, capturing temporal changes in forest structure.

**Study area:**

Estonia, hemiboreal forest zone. Field sampling was conducted between 2006 and 2024.

**Data coverage:**

The dataset comprises detailed stand-structure measurements from >600 forest stands, mostly represented by 2-ha plots. It includes most common hemiboreal forest types along gradients of dominant tree species and soil moisture. These range from low-productivity, dry Scots pine–dominated forests to black alder–dominated mobile-water swamps, with particularly strong representation of productive Norway spruce–deciduous mixed forests and drained peatland forests. Stand ages range from a few years post clearcutting to old stands with the dominant tree layer >200 years old. Old stands without signs of management are well represented (98 study stands) and constitute a substantial reference set for naturally developing and old-growth forest conditions in the region. The stands encompass a broad range of silvicultural treatments, including post-clearcut and post retention-cut succession, mature forests with sanitary cutting history, stands subject to precommercial and commercial thinning, and shelterwood cutting. In addition, a subset of stands includes repeated measurements of forest structure following ditch closure (rewetting) as part of a peatland forest restoration experiment.

**Methods:**

Live trees, standing dead trunks and stumps were recorded along strip plots (area-based sampling), while downed dead wood items were measured along transects (line-intersect method).

**Applications:**

The dataset enables modelling above-ground tree carbon, quantitative assessment of habitat conditions for biodiversity, and analyses of their relationships with forest characteristics. It enables analyses of how specific forest management practices (clearcutting, retention forestry, shelterwood harvest, thinning) affect the stand structure, habitat quality, and carbon stocks. Because the dataset includes geopositioned measurements collected starting from the early 2000s onward, it can be combined with time-matched or contemporary remote sensing data, or with newly collected field data from stands of comparable ages, to assess structural changes in similar forest types over time. Given that hemiboreal forest types share structural features with closed-canopy boreal and northern temperate forests dominated by spruce–pine–deciduous mixtures, the dataset is applicable for regional modelling across approximately ten degrees of latitude, spanning from the Fennoscandian middle-boreal zone to the northern limit of the beech region in mid-Lithuania.

Specifications Table

**Table:** 

Subject	Biology
Specific subject area	Forest structure data from >600 hemiboreal forest stands representing a variety of forest site types, management histories, and successional stages.
Type of data	Raw data (quality assessed), Processed data: excel files; Geospatial data: ESRI Shapefiles
Data collection	Data were collected during multiple field campaigns between 2006 and 2024 using a combination of area-based sampling (for live trees and snags) and line-intersect methods (for downed dead wood items). Measurements were conducted along pre-established transect lines within each stand. For live trees and snags, diameter at breast height was recorded; for downed dead wood, diameter was measured at the point where it intersected the transect. Tree species was identified for all live and dead trees, and decomposition stage was recorded for dead wood.
Data source location	Country: Estonia (57.3–59.5 latitude; 21.5–28.1 longitude). The data are accompanied with a map layer with stand polygons.*.*
Data accessibility	Repository name: Zenodo Data identification number: 10.5281/zenodo.18271070 Direct URL to data: https://doi.org/10.5281/zenodo.18271070
Related research article	Diverse research articles*.*

## 1 Value of the Data

• The dataset offers tree, transect and stand-level forest structure measurements, enabling flexible aggregation and calculation of wide range of metrics (e.g., woody species diversity, relative proportion of dead wood of total tree volume). Data can be summarized by tree species or other attributes (e.g., diameter, decay class), and include standard stand-level metrics such as live and dead wood volume per hectare. Stand ages linked to the measurement year allow age-structured modelling and comparisons among stand successional stages.
• A map layer with stand polygons allows users to link each field-measured stand to airborne and satellite data from the same inventory year, supporting spatial modelling and calibration of remote-sensing-based estimates of stand structure, woody biomass or habitat indicators.
• The dataset provides high-resolution measurement of dead wood, a structural component that cannot be reliably quantified using remote sensing. It includes detailed records of standing and downed dead wood, including (in a subset of stands) fine woody debris down to 3 mm in diameter. Such a resolution is not available in common large-scale sampling schemes (such as national forest inventories), making these data valuable for studies on habitat availability, decomposition processes, carbon pools, and biodiversity associated with dead wood.
• The data can be used to assess how different forest management practices correspond to variation in aboveground timber and carbon stocks, stand structure, and biodiversity-relevant habitat elements. Because the dataset includes stands with known histories of clearcutting, retention forestry, shelterwood harvest, sanitary cutting, and thinning, users can compare structural outcomes associated with commonly applied management regimes, including benchmarking against old-growth forest conditions.
• The temporal span of the dataset enables reconstruction of past stand structural conditions (e.g., in relation to changing forestry practices or climate). Measurements beginning in the early 2000s provide a basis for establishing historical reference points, supporting studies that need background data for long-term monitoring, repeated inventories, or retrospective analyses.
• The dataset is applicable for regional modelling across a broader latitudinal gradient where boreal and temperate forest structures overlap. Because hemiboreal forests share structural characteristics with both southern to middle boreal and northern temperate forest types, the data can be used in regional analyses spanning approximately ten degrees of latitude, from the Fennoscandian middle-boreal zone to the northern limit of the beech region in mid-Lithuania.

## 2 Background

Stand structure is a defining characteristic of forests, central in their ecological and habitat description, and for estimating the effects of conservation and management practices [1]. Because trees hold substantial aboveground carbon, data on stand structure—including the characteristics of live stand and dead wood—are also essential for estimating carbon stocks [2]. In European hemiboreal region, such estimations are complicated by a patchy forest landscape where structural attributes vary with successional stage, management history, soil conditions, and dominant tree species [3]. These considerations motivated the sampling of stand structure data across a wide array of hemiboreal forests, now assembled into this dataset.

Over the past two decades, subsets of these data have been used in studies (i) quantifying old-growth structural baselines [4], and describing post-harvest dead wood pools and their dynamics [5]; (ii) as a background data in diverse biodiversity studies [e.g. [6], [7], [8], [9], [10]]; (iii) describing long-term drainage impacts in naturally wet and drained peatland forests [10]; and (iv) evaluating how different forest management practices correspond to timber yield, aboveground carbon and habitat availability [11,12]. By consolidating previously independent datasets, including material not published earlier, this dataset provides a unified resource for broader ecological and modelling applications.

## 3 Data Description

**Fig. 1:**
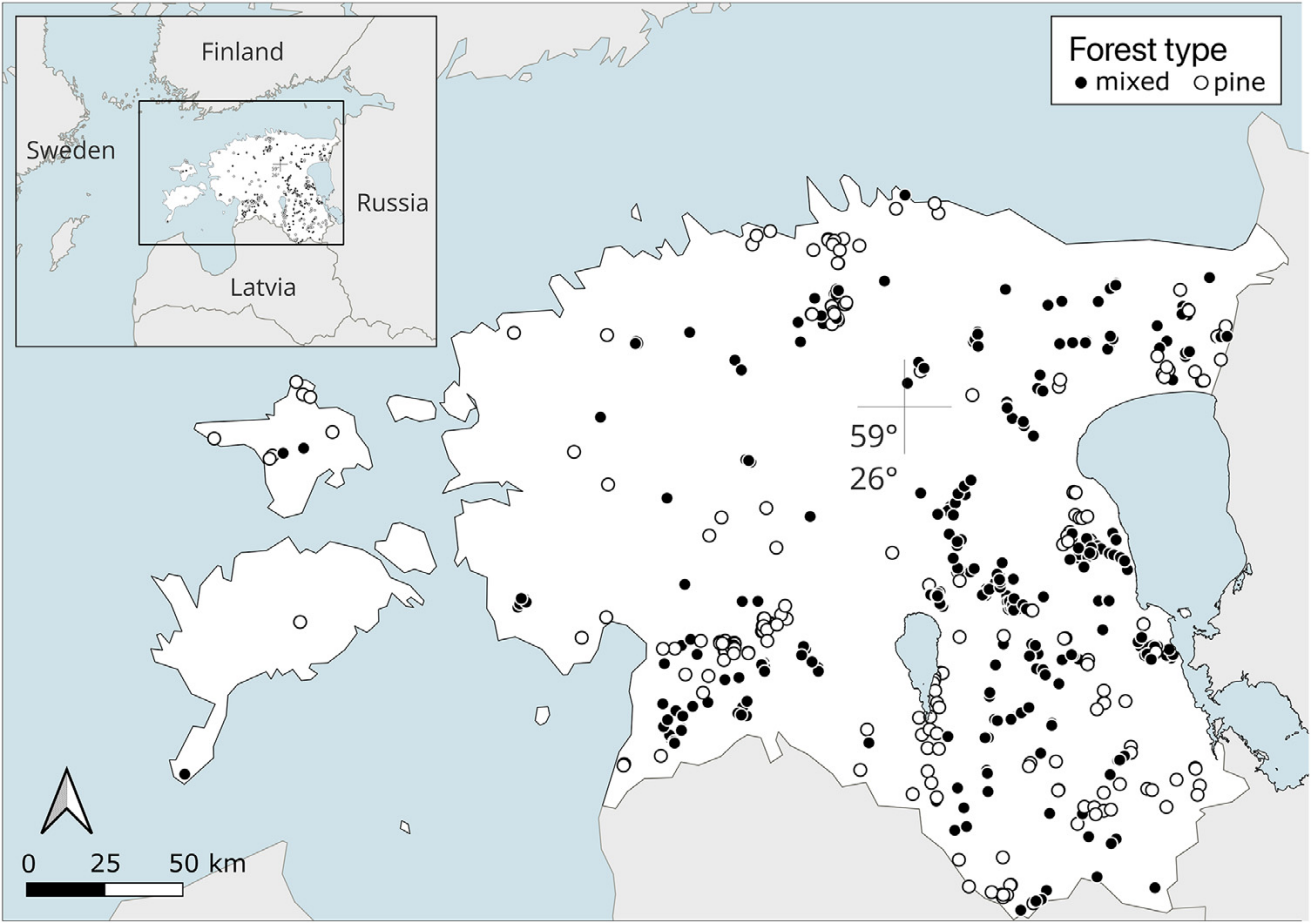
The locations of studied forest stands in Estonia.

This article describes the dataset available in the Zenodo repository [13], which contains forest structure data from Estonian hemiboreal forests across different forest types, successional stages, and forest management backgrounds (Table 1). The data were collected in >600 forest stands between 2006 and 2024 (Fig. 1). The repository includes a README file in (txt format), one GIS map layer, and four clusters of csv files. An overview of the contents of the README and csv files is provided in Table 2.

**Table 1:** Overview of the studied forest types, successional stages and forest management treatments across the different subsets of the data. The standardized survey method allows direct comparisons across the subsets.

Subset	Site types	Successional stage	Management	No. of stands	Study year	Subset name	References
1	Dry boreal, Meso-eutrophic, Eutrophic, Swamp, Drained peatland	E, Mi, Ma, O	clearcut, green tree retention, commercial thinning, slash harvesting	148	2006–2023#	LIST	[4,5,10]
2	Drained peatland: Myrtillus	Mi, Ma	ditch closure (rewetting), commercial thinning, gap harvesting, understory removal	65	2014, 2018, 2022##	DREX	[14]
3	Meso-eutrophic, Eutrophic, Drained peatland	Ma, O	protection for an iconic bird species	20	2014	CICNIG	[15]
4	Dry boreal, Meso-eutrophic, Eutrophic, Paludifying, Swamp, Drained peatland	Ma, O	edge effects: thinning and clearcuts in adjacent stands	101	2019	RMK forest fragments	[12]
5	Eutrophic	Mi	precommercial thinning	18	2022–2024	TREC	
6	Meso-eutrophic, Eutrophic, Drained peatland	Mi, Ma, O	sanitary cutting	19	2009		
7	Meso-eutrophic, Eutrophic	Mi, Ma, O	commercial thinning	59	2018–2019	RMK spruce forests	[11]
8	Meso-eutrophic, Eutrophic	Ma	edge effects: clearcuts in adjacent stands	10	2017		
9	Dry boreal, Meso-eutrophic	Ma	shelterwood harvesting	90	2011–2015###		[16]
10	Euthropic, Swamp, Drained peatland	E, Mi, Ma, O	sanitary cutting	24	2024		
11	Dry boreal, Paludifying	E, Mi, Ma	after fire: natural regeneration, salvage logging	9	2010–2015*		[17]
12	Meso-eutrophic, Eutrophic	E, Mi, Ma, O	planting vs. natural regeneration	40	2020	LaSa	[18]
**TOTAL**				**603**			

The abbreviations for successional stages are: E, early successional; Mi, middle aged; Ma, mature; O, old.

# a subset of 71 stands studied twice with a 12–18-year time gap, 3 stands studied three times.

## a subset of 41 stands studied three times: before and twice after the management treatments.

### a subset of 20 stands studied twice: before and after the management treatments.

⁎ a subset of 6 stands studied twice with a 5-year time gap.

**Table 2:** Content of the text and data files of the dataset (DOI: 10.5281/zenodo.18271070).

Table	Title	Content
0	README.txt	Overview of the dataset and repository structure. Describes all tables and explains linking keys used across tables. Also describes the GIS data, including geometry type, coordinate reference system and projection, and the identifiers used to link spatial features to tabular data.
1_subsets_stands_transects (four csv files)		
1.0	metadata_subsets_stands_transects	Describes all variables defines terms and abbreviations, specifies measurement units in Tables 1.1, 1.2 and 1.3
1.1	subsets	Overview of the original studies contributing to the dataset, including subset ID, study period, sampling design, and the successional stages and forest management conditions covered. This table expands the information summarised in Table 1.
1.2	stands	Data for each study stand, including stand ID (linked to the GIS map layer), stand area, stand successional stage and age at the time of sampling, forest site type, centroid coordinates, study year, and survey effort. Survey effort is reported as the number and total length of transect lines (used for dead wood measurements) and strip area (used for live tree measurements). For stands inventoried on multiple occasions, survey effort is reported separately for each inventory year. This table also contains stand level estimates of dead wood and live tree volumes scaled to per-hectare values.
1.3	transects	Data for each transect line within study stands, including transect ID (linked to the GIS map layer and stand ID), position within the stand, transect length, and associated strip area. This table also contains transect-level estimates of dead wood and live tree volumes scaled to per-hectare values.
2_standing (two csv files)		
2.0	metadata_standing	Describes all variables, defines terms and abbreviations, specifies measurement units in Table 2.1
2.1	standing	Records of measured live trees, snags and stumps associated with each transect line, including transect ID, tree species, and diameter at breast height. For live trees and snags, the height and estimated volume is given. For snags and stumps, decay stage is given.
3_CWD (two csv files)		
3.0	metadata_CWD	Describes all variables, defines terms and abbreviations, specifies measurement units in Table 3.1
3.1	CWD	Records of individual downed coarse woody debris (CWD) items intersecting transect lines, including transect ID, tree species, diameter measured at the point of intersection, decay stage, and CWD subtype (e.g. fallen trunk, sawn log, root, branch). For definitions see Fieldworks.
4_FWD (two csv files)		
4.0	metadata_FWD	Describes all variables, defines terms and abbreviations, specifies measurement units in Table 4.1
4.1	FWD	Records of individual downed fine woody debris (FWD) items intersecting transect lines, including transect ID, tree species (where identifiable), diameter measured at the point of intersection, and decay stage. FWD measurements are available for a subset of study stands only. For definitions see Fieldworks.

The dataset is organised into three data levels: study stand, transect, and individual object. Stand-level data provide aggregated metrics for each study stand based on the underlying transect and object-level records. Transect-level data include summaries of measurements aggregated for each transect line within a stand. The individual-object level contains records for each measured live tree, snag, stump or dead wood item, including attributes such as tree species, diameter, and decay stage.

## 4 Experimental Design, Materials and Methods

### 4.1 Study region

Estonia is a lowland country, which is situated in the centre of the European hemiboreal zone at the coasts of Baltic Sea; it has a west–east gradient from maritime to subcontinental conditions [3]. The mean air temperature is 18 °C in July and −3 °C in January, and the average annual precipitation is 600–700 mm (1991–2020 data). The topography is of glacial origin; it is characterised by flat and undulating moraine plains as well as glaciolacustrine plains with abundant clayey deposits and extensive post-glacial paludification. Approximately half of the country (total land area 45,339 km²) is forested; however, following a long history of land use only about 2 % of this consists of old natural stands [19]. Production forestry on permanently or temporarily waterlogged soils relies largely on artificial drainage (the establishment and maintenance of ditch networks). Approximately 25 % of the Estonian forest area is drained, with a mean ditch density of 4.5 km⁻² (based on the Estonian base map). Historical draining is common even within currently protected areas. 85 % of the forest stands studied in this dataset were situated in continuous forest land (i.e., areas that have remained forested over the past century); the remaining stands were, a hundred years ago, either mires with sparse tree and shrub cover or seminatural grasslands.

### 4.2 Studied forest types

Six common natural groups of forest site types arranged along soil richness and moisture gradients [20] are represented in this dataset:

1. Dry boreal (*N* = 154 stands). Mostly *Vaccinium*-type forests on higher fluvioglacial landforms and till mounds with Podzols (pH 3.5–5.0), where water reaches the soil surface only sporadically and the top layer is periodically dry. The stands are dominated by Scots pine (*Pinus sylvestris*) and typically yield 250–350 m³ ha⁻¹ of timber at rotation age. During post-clearcut succession, they develop a continuous moss cover (occasionally lichen cover), a species-poor undergrowth (mostly *Vaccinium* and *Calluna* shrubs), and a very sparse understorey. This group also includes some stands in heath forests (*Calluna* and *Cladina* types), representing drier and more nutrient-poor conditions, as well as poor paludifying stands (*Uliginosum* and *Polytrichum*–*Myrtillus* types), which are equally nutrient-poor but wetter; both remain structurally and functionally similar to *Vaccinium*-type forests.
2. Meso-eutrophic (*N* = 158 stands). Mostly *Oxalis*-type forests on till mounds or rolling plains with Podzols or Stagnic Luvisols (pH 3.2–4.2), where groundwater lies deeper than 2 m. Naturally regenerated stands are conifer–deciduous mixtures that, depending on soil productivity, contain either Scots pine or Norway spruce (*Picea abies*), and yield timber volumes up to 800 m³ ha⁻¹. The understorey is sparse to moderately abundant, and the undergrowth is relatively species-rich with few or no shrubs. In mature and old stands, the moss cover is continuous and moderately thick. In commercial forests, Norway spruce is preferred for planting, those stands tend to have highly depauperate ground vegetation.
3. Eutrophic boreo-nemoral (*N* = 108 stands). Mostly *Aegopodium*-type forests, predominantly on undulating sandy till plains with favourably moist (and in springtime anaerobic) Gleyic Cambisols or Luvisols (pH 4.7–6.5) and almost no organic horizon. Naturally regenerated stands become mixtures of deciduous pioneer species—birches (*Betula* spp.), European aspen (*Populus tremula*), and grey alder (*Alnus incana*)—and Norway spruce; they yield up to 700 m³ ha⁻¹. Both the understorey and undergrowth are species-rich and range from sparse to dense depending on canopy closure, while the moss cover is sparse and fragmented.
4. Eutrophic paludifying (*N* = 40 stands). Mostly *Filipendula*-type forests on flat, lowland areas with wet soils, predominantly Gleyic or shallow peat-gleys with 11–30 cm thick moder or moder-humus forest floors, developed under the influence of weakly mobile, carbonate-rich groundwater. Some eutrophic paludifying stands have developed following the drainage of very shallow fen soils. All eutrophic paludifying stands have relatively high soil acidity and high humus content. The tree layer consists mainly of birch and other deciduous species, while stands influenced by drainage can also be dominated by Norway spruce. Typical stock volumes are 200–226 m³ ha⁻¹. The understorey and ground vegetation are species-rich, reflecting high nutrient availability, and dominated by tall herbs and broadleaved species, while the moss layer is typically fragmented.
5. Swamp forests (*N* = 31 stands). Mostly mobile-water swamp forests in lowlands and valleys along rivers or around bogs. Their thin Eutric Histosols and Fluvisols (pH 5.0–6.5) are frequently flooded; the water level drops 20–40 cm below the ground surface only during prolonged drought periods. On thinner peat horizons, black alder (*Alnus glutinosa*) characteristically forms alder carrs, while birches dominate where peat horizons are thicker and water is more stagnant. Typical stock volumes are 150–200 m³ ha⁻¹. The abundance of the understorey varies widely, and the undergrowth is species-rich and patchy due to the highly irregular microrelief.
6. Drained peatland forests: *Oxalis* (*N* = 39 stands) and *Myrtillus* (*N* = 73 stands) types stands drained for forestry. The *Oxalis*-type stands are dominated by Norway spruce and have well-decomposed peat soils (pH 4.0–6.5), representing residuals of the original swamps or fens; the current tree layer was established after drainage, with ditching effects persisting for >50 years. The shrub layer is sparse to moderately sparse. The *Myrtillus*-type stands develop on thicker (bog) peat deposits and are dominated by Scots pine, with varying proportions of birch and Norway spruce. The shrub layer is sparse to moderately sparse, while dwarf shrubs such as *Vaccinium myrtillus, V. uliginosum, V. vitis-idaea,* and *Rhododendron tomentosum* are common. The ground layer is patchy, with patches frequently dominated by both mosses and grasses.

### 4.3 Studied stand successional stages and designation of natural reference stands

In this dataset, stand successional stage is classified according to commonly used hemiboreal forest development stages. Early-successional stands are defined as those up to approximately 30 years after clear cut. Age thresholds for subsequent stages vary by forest type and site productivity: in Scots pine-dominated stands, middle age is defined as 30–110 years depending on productivity class, and maturity begins at 80–110 years, while in spruce–deciduous mixed stands, middle age typically starts from 30 years and maturity begins at 60–80 years (Table 3; [21]). Based on these definitions, the dataset includes stands from all major successional stages, with old stands being the most represented group (*N* = 233; Table 3). Early successional pine dominated stands are comparatively underrepresented (*N* = 29; Table 3), reflecting the focus of the original studies that contributed to the dataset. In addition to stand successional stage, we identify a subset of old stands that can be considered natural and used as reference points for forest structure under unmanaged conditions. Because some old stands show clear signs of management (e.g. stumps), only 98 of the 233 old stands were classified as natural.

**Table 3:** Stand development stages in relation to stand age (years) and site productivity class.

Site productivity class	E	Mi	Ma	O	TOTAL studied
Scots pine dominated stands (Dry boreal and *Vaccinium myrtillus* drained peatland)					
0	0–29 (1)	30–79 (0)	80–109 (2)	>110 (2)	**5**
1	0–29 (6)	30–79 (2)	80–109 (9)	>110 (18)	**35**
2	0–29 (14)	30–79 (23)	80–109 (13)	>110 (49)	**99**
3	0–29 (8)	30–89 (31)	90–119 (17)	>120 (40)	**96**
4	0–29 (0)	30–99 (6)	100–129 (6)	>130 (11)	**23**
5	0–29 (0)	30–109 (1)	110–139 (0)	>140 (1)	**2**
**TOTAL studied**	**29**	**63**	**47**	**121**	**260**


Norway spruce–deciduous mixed stands (Meso-eutrophic, Eutrophic boreo-nemoral, Eutrophic paludifying, Swamp and *Oxalis* drained peatland)					
0	0–29 (18)	30–59 (21)	60–89 (12)	>90* (10)	**61**
1	0–29 (39)	30–59 (19)	60–89 (49)	>90* (30)	**137**
2	0–29 (29)	30–69 (12)	70–99 (18)	>100 (37)	**96**
3	0–29 (5)	30–69 (3)	70–99 (3)	>100 (30)	**41**
4	0–29 (2)	30–79 (0)	80–109 (1)	>110 (5)	**8**
**TOTAL studied**	**93**	**55**	**83**	**112**	**343**

In the Estonian forestry system, site productivity classes range from 0 (most productive) to 5 (least productive). The abbreviations for successional stages are: E, early successional; Mi, middle-aged; Ma, mature; O, old. The number of studied stands in the consecutive groups is given in parentheses.

⁎ In spruce dominated stands >100.

### 4.4 Studied forest management practices

In Estonia, forest management for timber production is based on native tree species. Operationally, it follows an even-aged, clear-cutting-based silvicultural system in which cutovers were earlier normally left for natural regeneration but are since mid 20-th century mostly regenerated by planting or sowing conifers (Norway spruce and Scots pine), and subsequent management includes multiple thinning episodes. Accordingly, the early-successional stands in the dataset mostly originate from clearcutting, with or without green-tree retention. Most of the early-successional data were collected in 2005–2007, when harvest residues (slash) were generally left on site after clearcutting, with an exception of a small set of stands sampled in 2010 [5]. In contrast, the current practice includes much more frequent slash removal, now carried out on approximately one-third-of cutovers [22].

The management backgrounds are most variable in the middle-aged and mature stands, which originate either from planting or natural regeneration [4,10,11]. Specific datasets from managed forests focus on the relationships between forest structure and commercial thinning [11], shelterwood harvesting [16], edge effects from clearcuts adjacent to the sampled stands [12], and rewetting (ditch closure) for wetland forest restoration purposes.

### 4.5 Fieldwork

The primary sampling unit was the forest stand, as delineated in the Estonian Forestry Registry. It refers to a forest landscape unit that has a more or less uniform site type, stand age, and overstorey tree species; it is also the management unit in even-aged silviculture.

**Fig. 2:**
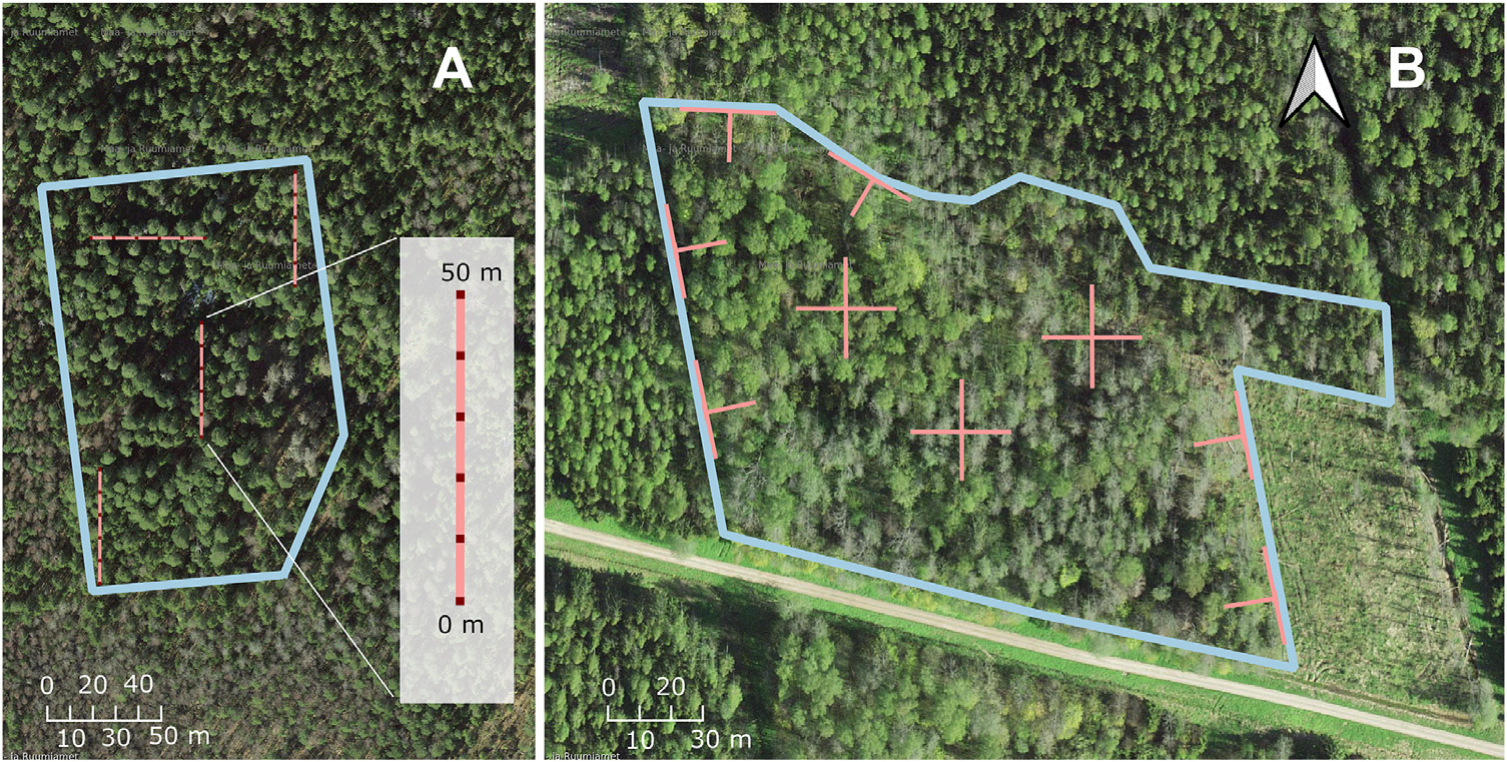
Two examples of sampling setups for forest structure at the stand scale. **A.** Placement of four straight 50-m transects within a stand. Three north–south transects are placed at the centre and near the northern and southern edges of the stand. Along each transect (small scheme on the right), CWD was recorded on the transect line, live trees within 2 m of the line, and snags within 5 m; additionally, 1-m sections were established every 10 m to sample FWD. **B.** Placement of nine crossed transects within a stand. Three full crosses (total transect length 60 m) were installed in the interior of the stand, and three half-crosses (total length 45 m) near the boundaries adjacent to stands of different ages. CWD was recorded along the transect line. In this example, live trees and FWD were not mapped.

In most subsets of the dataset, measurements were taken along transects (sampling lines) within a 2-ha plot in the stand; a standard plot size was important to represent similar extent of spatial variability and to relate to biodiversity (that depends on spatial scale) (Fig. 2). Rectangular plots were preferred, although landscape features occasionally required more complex shapes. In 56 % of studied stands, the entire stand was surveyed. Prior to the fieldwork, the positions of transects were determined on GIS map layers. Within each stand, one to five straight (Fig. 2A) or crossed (Fig. 2B) 50 or 60 m transects were established, depending on a study. The number and arrangement of transects varied by forest type, with denser sampling in structurally poor dry boreal and drained stands. The exact sampling scheme and total transect length for each stand are provided in data tables uploaded to Zenodo (Table 1.3). Each transect was then marked in the field, and forest structure was recorded using a combination of the line-intersect method for coarse woody debris (CWD) and fine woody debris (FWD; measured in a subset of stands) and plot-based measurements for standing trees. In all cases, the sampling effort was sufficient to estimate per-hectare values for the recorded structural parameters.

Within each transect, four categories of structural elements were recorded: live trees, downed coarse woody debris (CWD), downed fine woody debris (FWD), and snags (standing dead trees with or without broken top).

(i) Live trees. The diameter of all live trees within 2 m on both sides of the transect line was measured at breast height (DBH). As a rule, all trees with DBH ≥10 cm were included. An exception was made for datasets addressing young forests and the forests of the drained peatland type: because the dominant tree layer in these forests included trees with DBH slightly below 10 cm, trees with DBH ≥5 cm and ≥7 cm were respectively included in these datasets. Diameter measurements included bark.

(ii) Downed coarse woody debris (CWD). The diameter (with attached bark) and decay stage of each CWD item were determined at the point where it intersected the transect line along the entire length of each transect. All items with a diameter ≥10 cm at the intersection point were included. In addition to fallen trunks and branches, CWD included sawn pieces (“logs”) typical of recently clearcut or thinned stands, exposed dead roots, woody material buried in the litter or moss layer (but not in soil), and submerged wood in flooded forests.

(iii) Downed fine woody debris (FWD). The diameter and decay stage of each FWD item were measured in six 1-m sections placed at 10-m intervals along each transect. All items with a diameter of 0.3–9.9 cm at the intersection point were included. This category comprised small stems, branches, and woody remains of shrubs. All intersecting pieces were carefully dissected from the litter or moss layer to measure their exact diameter at the point of intersection. FWD was measured in approximately half of the studied stands.

(iv) Snags. The DBH of all snags was measured within 5 m on both sides of the transect line. Dead trees with or without a broken top were included if they were ≥1.0 m tall (in the case of broken-top snags) and had DBH or top diameter ≥10 cm (≥5 or ≥7 cm in drained peatland forests and young forests, respectively). For broken-top snags, height was also measured to facilitate volume estimation. Diameter measurements included bark.

(v) Stumps. Cut or natural stumps (≤1.0 m tall) were included if they had top diameter ≥10 cm (≥7 cm in drained peatland forests). With some exceptions, the top diameter and height of stumps were measured within 5 m on both sides of the transect line. Stump counts ans measurements are available in approximately 50 % of the studied stands.

Decay stages of CWD were classified following [23], and those of FWD following [4]. For items in advanced decay (class V), diameter measurements were approximate due to collapse, fragmentation, and extensive overgrowth. Moss was removed from the sides of each item until decayed wood became visible.

### 4.6 Data treatment

The dataset provides both unit-level measurements (including measurements and secondary attributes such as species for each live tree, CWD item, FWD item, snag or stump, and decay stage for all dead wood items) and per-hectare volumes of CWD, snags, and living trees at both transect and stand level, facilitating future use of the data. Trunk volumes of live trees and snags were estimated using species-specific diameter functions [24] applied in practical silviculture in Estonia. For that, height of live trees was calculated based on model trees in study stands or estimated based on measured DBH and site productivity class [25]. The volume of CWD was estimated assuming circular cross-sections [26].

## Limitations

This dataset has two main limitations. First, it represents some hemiboreal forest conditions more comprehensively than others. This limitation should be considered when aiming to characterise overall hemiboreal forest structure or dead wood pools at the landscape or regional scale. Specifically, the 20–60 years age class is underrepresented relative to its current regional prevalence. Also, certain hemiboreal forest types have been less frequently sampled in the underlying studies, particularly Scots pine–dominated extreme-wet (bog forest) and extreme-dry (heath forest) types.

Second, the surveys of downed fine and coarse woody debris were designed for volume estimation, which cannot be readily translated into the number of individual trunks. Given sufficient transect length, the line-intersect method provides reliable estimates of dead wood volume across size fractions [27]. However, because a trunk may intersect a transect at any position along its length, the number of intersections of a given diameter cannot be directly translated into the number of fallen trunks per size class.

## Ethics Statement

The authors have read and follow the ethical requirements for publication in Data in Brief and confirm that the current work does not involve human subjects, animal experiments, or any data collected from social media platforms.

## CRediT Author Statement

**Raul Rosenvald:** Conceptualization, Formal analysis, Investigation, Data Curation, Writing - Review & Editing, Vizualisation; **Asko Lõhmus:** Conceptualization, Methodology, Investigation, Writing - Original draft; Funding aquisition; **Raido Kont:** Investigation, Writing - Review & Editing; **Ann Kraut:** Investigation, Writing - Review & Editing; **Piret Lõhmus:** Investigation, Writing - Review & Editing; Funding aquisition; **Liina Remm:** Investigation, Writing - Review & Editing; **Triin Tekko:** Investigation, Writing - Review & Editing; **Maarja Vaikre:** Investigation, Writing - Review & Editing; **Kadri Runnel:** Conceptualization, Investigation, Writing - Original draft, Project administration, Funding aquisition.

## Acknowledgments

We thank the researchers and field assistants who have contributed to the collection of these data over the years, in particular: Gen Alev, Aleksei Andrianov, Leif-August Kirs, Anneli Palo, Artur Reila, Katrin Rosenvald, Siim Sellis, Erko Soolmann, Siim Štšjogolev, and Indrek Tammekänd. We also thank Boris Meldre for his help with the illustrations.

## Funding

This work was supported by 10.13039/501100005189Estonian Research Council [grants IUT21–04 to RR, ETF6457, ETF7402, SF0180012s09, ETF9051, IUT34–7 and PRG1121 to AL; ETF7987 to PL; PSG825 and TK232 to KR]; Estonian State Forest Management Centre [grants No. 8-2/T11081MIMK and 8T160148MIMK to RR, LLOOM13051 to AL; LLOOM08244 to PL, and LLTOM18470 to KR]; Estonian Environmental Investment Centre [projects 11594 and 16288 to AL]; Estonian Environment Agency (LLOOM15080 to PL); Estonian Environmental Board (LLTOM16048 and LLOOM10074 to PL), and European Union through the European Regional Development Fund (the Centre of Excellence FIBIR).

## Declaration of Competing Interest

The authors declare that they have no known competing financial interests or personal relationships that could have appeared to influence the work reported in this paper.

## Data Availability

ZenodoData from: Dataset on stand structure in hemiboreal forests across management histories and stand ages (Original data).
